# Yeast Cells in Microencapsulation. General Features and Controlling Factors of the Encapsulation Process

**DOI:** 10.3390/molecules26113123

**Published:** 2021-05-24

**Authors:** Giulia Coradello, Nicola Tirelli

**Affiliations:** 1 Laboratory for Polymers and Biomaterials, Fondazione Istituto Italiano di Tecnologia, 16163 Genova, Italy; giulia.coradello@iit.it; 2 Department of Chemistry and Industrial Chemistry, University of Genova, Via Dodecaneso 31, 16146 Genova, Italy; 3 Division of Pharmacy and Optometry, School of Health Sciences, University of Manchester, Oxford Road, Manchester M13 9PT, UK

**Keywords:** drug delivery, food technology, diffusion phenomena

## Abstract

Besides their best-known uses in the food and fermentation industry, yeasts have also found application as microcapsules. In the encapsulation process, exogenous and most typically hydrophobic compounds diffuse and end up being passively entrapped in the cell body, and can be released upon application of appropriate stimuli. Yeast cells can be employed either living or dead, intact, permeabilized, or even emptied of all their original cytoplasmic contents. The main selling points of this set of encapsulation technologies, which to date has predominantly targeted food and—to a lesser extent—pharmaceutical applications, are the low cost, biodegradability and biocompatibility of the capsules, coupled to their sustainable origin (e.g., spent yeast from brewing). This review aims to provide a broad overview of the different kinds of yeast-based microcapsules and of the main physico-chemical characteristics that control the encapsulation process and its efficiency.

## 1. Introduction

**Short recap about yeast**. Yeasts are uni/multicellular eukaryotic organisms, originally thought to be ascomycetous fungi, but later recognized to also comprise basidiomycetous organisms; more typically, yeasts reproduce asexually (rapid duplication) but can also adopt sexual reproduction. A consensus definition has been proposed, and identifies yeasts “as those fungi whose asexual growth predominantly results from budding or fission, and which do not form their sexual states within or upon a fruiting body” [1]. Yeasts are best known as fermentative fungi, but it is worth noting that they are also very adaptive organisms, and have evolved the capacity to thrive (and ferment) both under aerobic and anaerobic conditions [2]. As a further testament of their capacity of adaptation, they have shown the capacity to acquire resistance to bisulfite [3] or synthetic antifungals [4,5] to the point that some yeasts can even survive nearly saturated brine solutions (e.g., *Debaryomyces hansenii*, also known as *Candida farmata* [6]). The industrial importance of these microorganisms resides in their rich metabolic activity (the very word ‘enzyme’ derives from the Greek word for leaven, ζύμη (zýmē)), which since the dawn of time has been heavily employed by humans, predominantly in food processing. Among the most popular yeasts in this business area, one could mention *Saccharomyces cerevisiae* and *Schizosaccharomyces pombe* (baking, beer making [7]), *Saccharomyces bayanus* (previously *Saccharomyces uvarum*, wine production [8]), *Candida kefyr* (formerly *Candida pseudotropicalis*, of which *Kluyveromyces fragilis* is the sexual stage used in dairy industry, flavors and enzymes synthesis, [9] conversion of lactose to ethanol for biofuel production [10]), and *Cyberlindnera jadinii* (commonly known as *Candida utilis*, for food flavoring [11]). 

It is worth mentioning, however, that not all yeasts are beneficial; aside from the possibility for humans to develop allergic reactions [12], some yeasts are directly pathogenic such as *Candida albicans* (previously known as *Monilia albicans*; candidiasis in the gastrointestinal, reproductive and respiratory systems [13]) or *Cryptococcus neoformans* (a major source of lymphocytic meningitis [14,15]). 

Of specific interest for this review are yeast strains capable of intracellular accumulation of large amounts of lipids, which are known as oleaginous yeasts. They can accommodate both endogenous and exogenous lipids routinely up to 20% (in some cases reportedly much more) of their weight. Examples in the literature go back decades; for example, in the 50s *Cryptococcus lipofer* (also known as *Torulopsis lipofera*) was one of the first yeasts to be shown to accumulate large amounts of sterols, [16] while in the 70s, *Cutaneotrichosporon curvatum* (formerly known as *Candida curvata*) and *Cutaneotrichosporon cutaneum* (also known as *Basidiotrichosporon cutaneum*) were first used to biotechnologically produce food-grade oil from cheese whey [17]. We refer the reader to specialized reviews for both the biochemical mechanisms presiding over lipid accumulation [18] and for the specific applications of these oleaginous yeasts [19], which range from the production of bio-diesel to that of food oils (e.g., cocoa butter substitutes). In terms of the identity of these oleaginous yeasts, possibly the most commonly employed are *Cutaneotrichosporon curvatus* (until recently known as *Cryptococcus curvatus*) [20] and *Yarrowia lipolytica* (formerly known as *Saccharomycopsis lipolytica*) [21]; a list of yeast strains with large lipid content, and therefore potentially apt for these applications, can be found in a review by Boundy-Mills [22], but it is worth noting that metabolic engineering of selected yeast types (above all of *Y. lipolytic*) is increasingly employed [23], as opposed to the use of a wider variety of microorganisms. 

**Yeast cells as microcapsules—technological advantages**. Encapsulation allows for compounds of interest to be protected from a potentially aggressive environment, and released in an active form; in principle, the release occurs at specific locations and with a desired time law. A microcapsule does so at 1–1000 μm scale.

Dimensionally, yeast cells suit this definition well. For example, *S. cerevisiae* (baker’s yeast) cells most commonly range around 5–10 μm, always with a rather narrow size distribution; the upper end of dimensions in the yeast world is likely the *Blastomyces dermatitidis*, which can be as large as 40 μm [24]. Yeasts fit the definition also from a functional point of view; for a long time (1976 patent) [25], yeast cells have been known for their capacity to absorb large amounts of hydrophobes, and the process can be tuned to produce hydrophobe-loaded cells, i.e. yeast-based microcapsules (YBMCs). YBMCs have been applied to encapsulate poorly soluble actives such as flavoring agents [26], antioxidants [27], biocides (acaricides) [28], increasing their water dispersibility [29], but also providing mechanical protection thanks to the robustness of the yeast cell walls [30]. Actually, cell walls do not fulfill only a mechanical role; they are also the main barrier to both loading [31] and release. It is noteworthy that the latter (the release of actives) typically requires the presence of water, which means that in a dry state (during storage), encapsulated actives are retained with a higher efficiency [32], and release is better obtained upon wet heating [33]. Whether due to solely to these barrier properties, or maybe also to the inherent antioxidant properties of their constituents [34,35], encapsulation in yeast increases the thermo/oxidative stability of actives [36,37], considerably more than e.g. then using ‘standard’ encapsulating agents such as oligo/polysaccharides, e.g. maltodextrins [33], β-glucans [38] or modified starch, or cyclodextrins [39].

Some YBMC features can be perceived as societal advantages. Firstly, they have a sustainable and low-cost origin. Since viability of yeast is not crucial for encapsulation [31], under-utilized by-products such as spent yeast from beer making [40] can be employed. Secondly, they can be seen as safe and consumer-friendly products. Aside from the presence of yeast in virtually all bakery products, whole yeast cells, their lysates or selected components are commonly sold as super-food or flavoring agents.

The relevant literature on YBMCs is, however, poorly coherent to the point that a common parameter such as the encapsulation yield (EY%: the weight ratio between loaded compound and yeast mass) is randomly reported in relation to dry [41] or variably hydrated yeast [31,42]. A more fundamental source of variability is due to the yeast species, since different strains of the same organism might behave differently, and particular culture conditions/various levels of biological stress are likely to also have an effect. 

**YBMCs—commercial and medical interest**. After the first seminal patent in 1976, the encapsulation of lipophilic materials in yeast cells has been protected by patents in applications related to carbonless paper, [43], fabric softeners [44], or fragrances [45] in the textile industry, nicotine for smoking cessation [46], drug delivery targeted to mucosal membranes [47], plant extracts [48], and food flavors [49]. For a more extensive review of these patents, please refer to Paramera et al. [50]. YBMCs may also have effects directly relevant to healthcare, although it is often difficult to pinpoint the precise biomolecular mechanisms and differentiate them from inflammatory (foreign-body) responses. For example, yeast cells have been linked to anti-mutagenic activity, potentially synergic with chemotherapy [51]; this may be related to the activation of inflammatory cells, which recognize yeast cells [52], phagocyte them [53] (also when in the form of yeast particles [54]), and end up exerting protective effects as a result [55]. Yeasts have also been reported to be able to trigger the opening of tight junctions in epithelial models [56], suggesting their use to control permeation through gut barriers, but this effect may also be caused by yeast triggering an inflammatory reaction: for example, *C. albicans* is well known to damage epithelia upon entry [57,58]. The kind of inflammatory responses elicited by YBMCs is still unclear; for example, when loaded with antigens for vaccines [59,60,61], siRNA [62], or even nanoparticles [63], YBMCs have produced impressive immunosuppressive effects in vivo [64], even without the use of immunosuppressive drugs [65], reportedly through combined pro-apoptotic and immunomodulatory effects [66]. 

## 2. Yeast Cells and Their Barrier Structures

**Figure 1:**
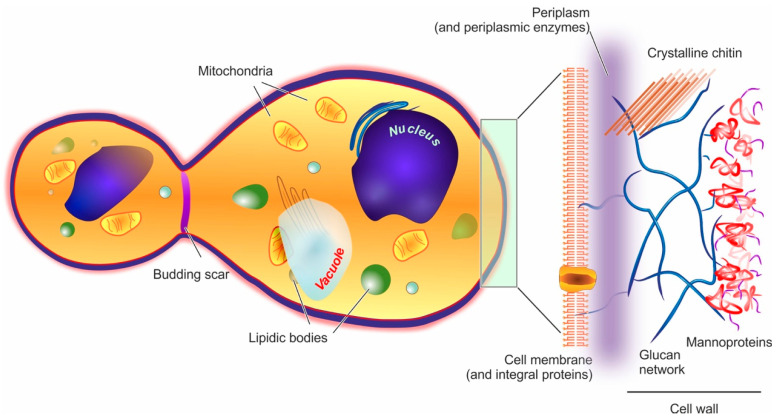
In the structure of a yeast cell (here represented in a post-budding state), the elements potentially acting as barriers to encapsulation (cell wall and cell membrane) are depicted in a magnified fashion on the right hand side of the figure.

Yeast cells feature an external cell wall and an inner membrane surrounding its intracellular environment, as shown in Figure 1. 

**Cell wall.** In yeast, the wall accounts for approx. 15–20% of the cell dry mass. Its thickness is very variable (70–200 nm), since it increases in response to compression or osmotic forces [67]; structurally, however, it is always a highly polar double-layered matrix (a kind of hydrogel). Its inner part is mainly composed of branched β-(1,3) and β-(1,6) glucans (about 50% of the overall wall) [68] hydrogen-bonded to 3–4% of mostly crystalline chitin [69]. This inner layer is likely to be the main contributor to the overall mechanical resistance of the whole cell wall [70]. The outer layer consists chiefly of mannoproteins, which are negatively charged proteins highly *N*- or *O*-glycosylated with mannose or mannosyl phosphate residues linked by 1,2-, 1,3-, 1,4- and 1,6-α-linkages (the latter mostly composed of short chains typically referred to as mannan) [71]. Mannoproteins are the main contributors to the cell wall surface properties, for example, the anionic mannosyl phosphate residues determine the yeast cell surface charge [72] and their reduction in number determines an increase in its hydrophobicity [73]. Furthermore, through their covalent linkage to the β-glucan layer, mannoproteins contribute to the wall outer porosity [74], and above all to yeast adhesion, for example, the *C. albicans* adhesins that allow its binding to oral epithelial cells are all mannoproteins [75]. 

Of note, the overall wall composition is rather constant throughout the different yeast species [76], and that of *S. cerevisiae* can be considered as representative for almost all Ascomycetes [77]; it must be pointed out, however, that although the main components are preserved, the actual detailed composition is not at all simple, with about 1200 genes having being linked to a role in the cell wall [78].

**Plasma membrane.** It is composed in equal parts of lipids (mainly glycerophospholipids and fatty acids, with a smaller quantity of sterols such as ergosterol and sphingolipids) and proteins, and is connected by glycoproteins and glycolipids to the cell wall, from which it is separated by a non-continuous, enzyme-rich region known as the periplasm. The membrane main functional role is the regulation of the transport from/to the cell. [79] In the outer part of the membrane, phosphatidylcholine, phosphatidylethanolamine, phosphatidic acid, and sphingolipids are mostly found, while the more negatively charged phosphatidylserine and phosphatidylinositol are mostly present only in the inner membrane [70]; as in mammalian cells, lipid rafts are commonly observed, and have important roles in protein segregation and localization [80], which in turn have crucial effects in cell growth and death [81], or mating and division [82].

**Intracellular ultrastructure.** The yeast interior is compartmented into a nucleus, one or, more commonly, several vacuoles, mitochondria, the endoplasmic reticulum, and a number of vesicular bodies that include peroxisomes and oxisomes. It is worth noting that vacuoles are large (sometimes similar in size to nuclei), mildly acidic (pH 6–6.5) vesicles, whose functions in both storage and stress response/digestion bear some functional analogies to mammalian lysosomes/autophagosomes [83,84].

## 3. YBMCs–One Name, Different Materials

**Figure 2:**
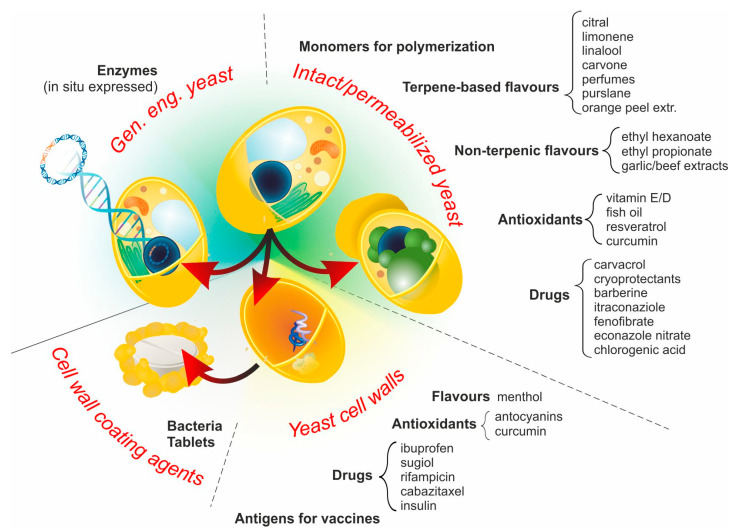
YBMCs can be grouped into four main classes, depending on whether intact or permeabilized cells, or their cell wall remnants are used for passive (diffusion-based encapsulation processes) or whether they are genetically engineered to produce one or more active compounds intracellularly. Examples of actives are provided for each class, and the corresponding references can be found in the text.

Four main approaches are employed to yield products that, although morphologically and functionally different, can be collectively referred to as YBMCs, as summarized in Figure 2. 

### 3.1. Intact Yeast Cells (No Pre-Treatment)

A large variety of hydrophobic compounds have been encapsulated in yeast, mostly in *S. cerevisiae* and predominantly for food applications. Intact cells have been employed for the encapsulation of terpenes (limonene, linalool and carvone [31]), perfumes [44], cryoprotectants [85], water-insoluble drugs (Itraconazole fenofibrate [86] and econazole nitrate [67]), orange peel essential oil [42,87], and antioxidants (α-tocopherol [88]). Although the process works in the absence of additional treatments, several comparative studies have demonstrated that encapsulation in intact cells is less efficient than that in plasmolyzed (see next section) ones; this has been shown with a variety of actives (e.g., purslane seed oil [89], cholecalciferol (vitamin D3) [90], barberine [91] and curcumin [92,93]). It is noteworthy, however, that the definition of ‘intact’ cells does not necessarily refer to the actual state of their barrier structures, for example, in the encapsulation of limonene in an inert-gas concentrated powder system, Errenst et al. used spent yeast from brewing processes, which is likely to have undergone significant autolysis [94], while in a previous study of ours, we used cells directly from different stages of culture (log or stationary phase) [31].

### 3.2. Permeabilized Yeast Cells

**Permeabilization through plasmolysis**. A number of compounds may not be able to cross plasma membranes, because poorly soluble in it due to high hydrophilicity, or ionic character. A common way to allow for the diffusion of such water-soluble compounds is that, prior to the exposure to the compound of interest, cells are subjected to a ‘plasmolysis’ i.e., a treatment that disrupts or weakens cell membranes; of note, plasmolytic treatments affect the integrity not only of the plasma membrane, but also of virtually all cytoplasmic organelles [95]. Plasmolysis can be obtained through two main approaches. 

(A) moderate or severe osmotic shocks, typically using hypertonic NaCl; for example, encapsulation of chlorogenic acid [27] or Antarctic krill oil [96] has been accomplished after a 5% NaCl/54–55 °C/48 h plasmolysis, while that of curcumin [92,97] or cholecalciferol [90] (both in ethanol solution) instead using 10% NaCl (for the rest, identical conditions). Other than the previously reported ones, examples of compounds encapsulated with such methods include limonene [31,32], garlic and beef extracts [32], and resveratrol [29]. 

(B) Treatment with at least partially water-soluble organic solvents, such as ethyl acetate [41,98,99] (e.g., for fish oil encapsulation [41]). The patent literature often also reports the use of ethanol and toluene for this purpose. Some sources specifically prefer to limit the use of the term ‘plasmolysis’ to the organic solvent-based processes, referring to those based on NaCl as an accelerated autolysis [98], which somehow implies that the processes also include the liberation of digestive enzymes in the intracellular space. Of note, autolysis can also proceed spontaneously in a temperature-activated fashion with a peak of efficiency around 50–55 °C (higher temperatures may lead to inactivation of hydrolases); [99] in some studies, solvent-based pre-treatments have been performed under conditions that indeed also favor autolysis (e.g., 55 °C/pH 5/24 h) [41]. 

**Permeabilization through partial enzymatic degradation.** In other cases, encapsulation may fail because the molecules are ‘too large’ to permeate. This effect is due to the barrier properties of both cell membrane and cell wall, where rather than being a purely dimensional issue (a molecule physically larger than the mesh size of a porous medium), it should better be interpreted as a result of the entropy-induced decrease in solubility with increasing molar mass (the ΔS of solubility decreases with it). Therefore, permeabilization treatments intended to boost the encapsulation of both hydrophilic and hydrophobic macromolecules tend to affect both the wall and membrane. For example, enzymes such as adenylate kinase and pyruvate kinase can be encapsulated only if the membrane is extracted with a surfactant such as Triton-X (similarly, bovine albumin is encapsulated only with Tween-80 or sodium dodecyl sulfate [100]), but improvements are obtained if the cell wall is permeabilized with gentle zymolyase treatment [101]. Similarly, bovine albumin is unable to penetrate into intact yeast cells, but can be encapsulated upon permeabilization with Tween-80 or with sodium dodecyl sulfate [100]. The effect of (partial) enzymatic digestion of the wall may have a positive albeit mild effect on its own: the use of a glucanase/protease cocktail (Glucanex R200) appears to increase the encapsulation efficiency, similarly to spontaneous autolysis at 50 °C [41]. In one report, proteases are said to have a negligible effect [102], but in this study, the experimental part reports the use of protease inhibitors rather than the proteases themselves. In fact, *S. cerevisiae* partially depleted of its cell-wall β-glucans (waste product from food factory extraction) was successfully applied to encapsulate a terpene such as citral [26], non-terpenic flavoring agents (ethyl hexanoate and ethyl propionate [26]), and even an acaricide (carvacrol [28]). 

**Use of organic solvents during encapsulation.** As an alternative to the above, organic solvents may be added during encapsulation (as opposed to pre-treatment as before). The solvent should enhance the solubility of the compound both in the external environment and in the cell walls, while also contributing to the disruption of membrane integrity. This is, however, a trial-and-error approach, where benzyl alcohol (about 30% in water) has worked well for itraconazole encapsulation [86], whereas 50% *v*/*v* ethanol has not worked well for resveratrol [29] or curcumin [92], but pure ethanol was useful to encapsulate free radical polymerizable monomers, yielding styrene nanoparticles [103]. A noteworthy example is the encapsulation in (pure) DMSO, which both destroys the integrity of membranes and changes the polarity of the cell wall, allowing the diffusion of short hydrophobic polymers [104].

**Other treatments.** Autoclaving (121 °C, 20 min) [42] and bleaching (1% H_2_O_2_ at pH 13 for 4 h) [86] reportedly increase the encapsulation efficiency of some hydrophobes; by virtually affecting all cell components, these treatments should be considered as intermediate between a ‘simple’ permeabilization, and those considerably more drastic processes, reducing whole cells into merely their cell walls (see next section). 

The reduction of disulfide-bonded mannoproteins with agents such as β-mercaptoethanol [100,102] or dithioeritritol [104] has shown a negligible influence on encapsulation, despite conflicting anecdotal reports. 

It is also worth mentioning that freeze drying may also have an influence on the permeability of wall/membranes; the use of freeze dried yeast is possibly more common than that of ‘wet’ cells, and it is safe to assume that the permeability should increase as a result of the process [86]. However, to our knowledge, this effect has seldom been assessed, also due to the variable conditions of the process including the type and concentration of cryoprotectants. We therefore would be inclined not to consider freeze drying among the permeabilization treatments.

**General effects of permeabilization techniques on yeast.** By means of IR spectroscopy Shi et al. highlighted that under autolysis-promoting conditions (55 °C/36 h), all forms of plasmolysis—surfactants such as Triton X-100 or sodium dodecyl sulfate, ethanol or 5% NaCl—essentially determined the same relative losses of nucleic acids and proteins and increases in lipids [105]. Paramera et al. showed a sharp decrease in the lipid gel-to-liquid crystal transition (from 158 to around 130 °C) upon NaCl plasmolysis, [106], but it is worth pointing out that the transition temperature depends on the yeast species, water content, and growth stage, for example, Normand et al. reported a phase transition at 173 °C [107]. Last, no deformity or rupture of the cell wall was observed in SEM images of 10% NaCl plasmolyzed yeast [89], but 2% NaOH (border line between a plasmolysis and the production of cell walls increased the average pore size (likely of the cell walls) from 4 to 30 nm [100].

### 3.3. Yeast Cell Walls (YCWs)

These constructs were obtained by thermal and strongly basic treatment (e.g., 1 M NaOH at a temperature above 80 °C followed by 1 M HCl at 60 °C [108]), which allows for the solubilization and extraction of all membranes due to lipid saponification; this is possibly accompanied by a significant degradation of mannoproteins and a partial deacetylation of chitin in the cell wall, although to our knowledge, the latter point has never been investigated. By this aggressive approach, the only remnants of the original yeast cells are their cell walls, which at this point are predominantly reduced to their glucan components; since they are likely to be the least immunogenic constituents of the yeast cells, the YCWs have also been administrated in vivo (e.g., in cancer immunotherapy) [109]. Due to the harsh treatment, YCWs do not necessarily present an intact barrier, and indeed, even nanoparticles can be loaded inside them, provided that they can then be trapped inside (e.g., through electrostatic interactions (cationic nanoparticles—naturally negatively charged cell walls, [110,111,112] or anionic nanoparticles—cationized (poly(ethylene imine) (PEI)-treated) cells walls)) [111]. Indeed, retention of the encapsulated material is possibly the critical issue to solve in YCWs; in alternative to specific interactions of the payload with the cell wall material, drug-loaded YCWs can be sealed with in situ produced hydrogels (e.g., calcium alginate around rifampicin-loaded YCWs [113], chitosan/chondroitin sulfate multilayers around anthocyanin-loaded YCWs [114] or Poloxamer/Pluronic gels around insulin-loaded YCWs [115]). Another solution is to induce the precipitation of the active compound. For example, albeit with low efficiency, a scarcely water-soluble drug such as sugiol can be accumulated in YCWs by repeated cycles of exposure of YCWs to an organic solution (e.g., acetone) and subsequent drying [116]. The release of the active would then be controlled by its own solubility; for example, a moderately soluble molecule such as ibuprofen is rapidly released, reaching saturation within minutes [93]. A recent, noteworthy approach is the use of a Maillard reaction with maltodextrins to strengthen the product of partial proteolytic digestion of *S. pastorianus* (essentially its cell walls), then used to encapsulate ascorbic acid in a spray-drying process [117].

In terms of applications, YCWs are often considered as pharmaceutical carriers; despite their potentially lower immunogenicity in comparison to their parent cells, YCWs are often employed as an avenue to specifically direct a payload to inflammatory cells such as macrophages in vitro [110,113], or through oral administration to M cells/Peyer’s patches in the intestinal tract [110,111]. Of note, in vivo YCWs have been shown to cause no appreciable damage to liver, spleen, or kidney [65].

It is worth mentioning that YCWs can also be employed as coating agents. For example, a flour made of *S. cerevisiae* cell walls has been used as an intermediate coating layer in the encapsulation of bacteria such as *Lactobacillus acidophilus* [118] and *Bifidobacterium bifidum* [119] in calcium alginate, allowing for enhanced survival under the high temperature and osmotic conditions during spray drying. Acid-treated yeast cell walls have also been used to coat paracetamol tablets, slowing down its release via tuning the lag time of the process [120]. 

### 3.4. Genetically Engineered Yeast

In this approach, the natural yeast barriers are circumvented by having the cells producing specific (bio)molecules within their own body, an approach sometimes referred to as a ‘biodrug’ [121]. Since the 1980s, yeast is one of the favorite microorganisms—and the preferred eukaryote—for the expression of recombinant, exogenous, and in particular, human proteins [122,123,124], which in turn can intracellularly produce small molecules such as ascorbic acid [125] and antioxidant amino acids [126]. The engineered cells can then be used directly as delivery vehicles, typically with the administration in the gastrointestinal tract [127] where yeast cells (contrary to e.g., bacteria) have a relatively high stability and a long permanence time. For example, yeast has been engineered to co-express Jerusalem artichoke’s cytochrome P450 73A1 isoform (also known as cinnamate 4-hydroxylase) together with human or yeast P450 reductase, [128] and was then tested in artificial models of the gastrointestinal tract, where it remained viable for 12 h and maintained its exogenous enzymatic activity for up to 4 h [121,129]. Of note, a mixed approach of genetic engineering and diffusional permeation has also been attempted. Yeast has been engineered to express galactosidase, which remains entrapped into the cell wall after membrane extraction, as an alternative to matrix immobilization [101]. After treatment, low concentrations of Triton X-100 are used to allow for an enhanced membrane permeability, where the substrate can enter the cell, be modified, and remain entrapped inside the microcapsule.

## 4. The Controlling Variables of Passive Encapsulation

Hereafter, we will focus on YBMCs belonging to category 1 of the list above (intact cells), for which the encapsulation of actives is a passive (=diffusive, non-energy-dependent) process of permeation, be it based on the passage of individual molecules or (less likely) of larger masses of a material such as oil droplets. Most considerations are also likely to apply to permeabilized cells (category 2 of the list above); however, due to the diversity of treatments (autolysis, solvent-induced plasmolysis, surfactants etc.), any generalization is in this case almost impossible. 

**Figure 3:**
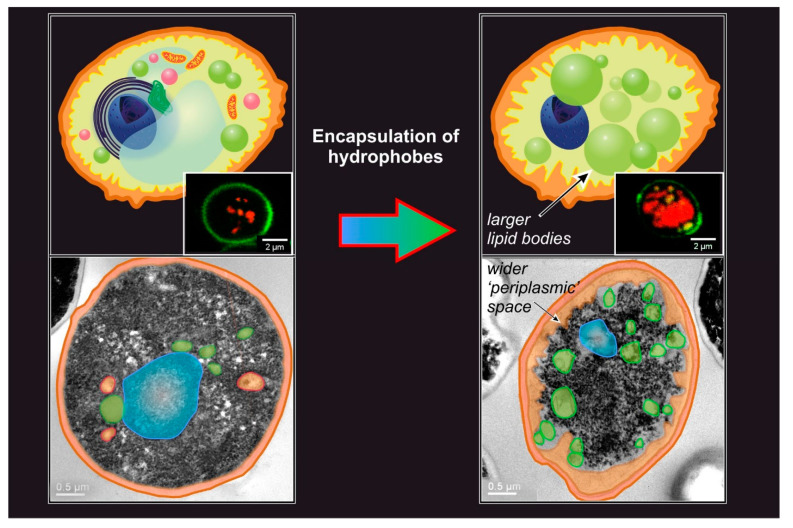
The yeast ultrastructure is dramatically altered by the encapsulation of hydrophobes; the TEM and confocal pictures show encapsulation in intact cells (limonene, 30% in relation to yeast dry weight, T = 60 °C, 4 h), but permeabilized cells behave similarly. The most striking result is the large increase in the number and dimensions of bodies that can be colored by the lipid stain Nile Red (in the insets, confocal images where the cell wall is stained by Concanavalin A, and Nile Red fluorescence highlights the lipidic bodies). However, as can be noted in the TEM images below, upon encapsulation of hydrophobes, the ‘periplasmic’ space (highlighted in light orange both in schemes and images) greatly increases; it is indeed difficult to define this area accurately, because the cell membrane has disappeared, but at this point, this is an area located between the wall and residual cytoplasmic content/lipid bodies (which localize predominantly in the center of the cell body, see inset on the right). Importantly, cytoplasm organization becomes more granular and—bar the lipidic bodies and the nucleus—hardly any organelle is recognizable. TEM images are courtesy of Federico Catalano, IIT Electron Microscope Imaging Facility (Genova, Italy).

The general outcome of passive encapsulation is a construct where the cell wall has remained largely intact, but the intracellular organization is dramatically altered. For example, typical features include the presence of a large number of lipid bodies, the loss of the usual intracellular ultrastructure, and although not commonly reported, an increase in the “periplasmic” space (Figure 3); for the latter, the word periplasmic is used to define an area immediately in contact with the internal surface of the cell wall, even if the cell membrane is no longer present.

Before the discussion of the individual factors controlling the encapsulation process, it is worth issuing two *caveats* in relation to the difficult comparison among the literature results.

1. The yeast strain, the composition of the medium used to culture it (or the brewing process, in case of spent yeast), and the growth phase the cells are in are all rather variable parameters, but their influence on the encapsulation process is essentially untested.
2. The analytical methods used to quantitate the processes also offer significant variability. For example, the amount of encapsulated hydrophobes is most commonly analyzed via HPLC or GC, but is sometimes obtained gravimetrically [41]; this analysis was performed after extraction of the yeast cells with organic solvents, but the variable nature of the solvent (e.g., Neobee [32], ethanol [91], methanol [31], methanol-chloroform [36], hexane, petroleum ether [89]) can be another source of variability. Last, before extraction, the cells are most often disrupted with bead mill disruption or via enzymatic digestion, with the former having been proven to be more efficient [31]. It is worth mentioning that a non-destructive and rapid quantification of hydrophobe encapsulation can be achieved via Nile Red lipid staining [31,130]. This method has some significant limitations: it requires an equilibration of at least 30 min [131] and a thorough calibration [31], which is critically necessary using different yeast strains (or other forms of microorganisms such as algae [132]) or operational conditions, since the results are quite dependent on cell morphology [133] and on whether an organic solvent was used as a carrier [134], leaving the fluorescence-free test as the most accurate approach [133].

Having therefore introduced the above *caveats* about quantitative comparisons, we hereafter discuss the key controlling factors of the encapsulation process.

**Hydrophobe/yeast ratio.** Several pieces of information are only seldom provided (e.g., the initial viability of the cells, whether the experiments refer to wet or dry masses of yeast, and the actual number of cells per unit of mass), but the most critical parameter to assess the reproducibility of the results is possibly the weight ratio between the compound to encapsulate and the yeast. This can strongly affect the two parameters used to evaluate the encapsulation process (i.e., encapsulation yield (EY%, the weight ratio between loaded compound and yeast) and encapsulation efficiency (EE%, the weight % of loaded compound in respect to its own initial mass)). For example, in the encapsulation of curcumin, it has been reported that with curcumin/dry yeast weight ratios between 0.2 to 1.4, EY% increased logarithmically, then slowed down and eventually reached a plateau at mass ratios higher than 3.3 [92]. It is worth pointing out a recurrent problem in literature: EY% is randomly reported against dry or wet yeast masses, which makes it hard to compare different studies; it would make considerably more sense to consider an encapsulation specific yield (ESY%) to selectively refer to the ratio with the dry yeast weight. Although in a slightly more hidden fashion, EE% also strongly depends on the hydrophobe/yeast weight ratio: if the amount of hydrophobe stays constant and that of yeast increases, the relative amount of encapsulated hydrophobe (i.e., the EE%) is naturally bound to increase. Importantly, a suitable hydrophobe/yeast may allow favoring EE% (more active encapsulated in absolute terms and less active wasted) over EY% (which would mean more heavily loaded yeast cells) [50], which may be important for high-value compounds.

**Temperature.** The fluidity of cell membranes can be sharply temperature-dependent due to the occurrence of a gel-liquid crystalline phase transition; for *S. cerevisiae*, this reportedly occurs around 30–37 °C [135], and the correspondingly higher membrane fluidity may explain the higher encapsulation efficiencies obtained at higher temperatures (up to 60 °C) [42,92].

**Pressure, vacuum, and electric fields.** To our knowledge, to date, only a handful of reports have used high pressures for encapsulation, which may be beneficial due to the induction of pores in the cell wall, as has been shown for shear forces or short pulsed electric field (PEF) [136]. However, there appears to be no conclusive evidence of improvements in encapsulation efficiency through the application of high pressure: Shi et al. used high pressures (25 MPa, 40 °C, 4 h) for chlorogenic acid [27] or resveratrol [29]), but no control at room pressure was employed; Errenst et al. employed a high pressure system where a limonene/water emulsion in CO_2_ was mixed with yeast carried by a nitrogen stream [94], but there the high pressure was an integral component of their process (=no control at low pressure would be possible). More recently, Dimopoulos et al. saw significant improvement in the speed of oregano oil encapsulation upon the application of high pressure (800 bar) or PEF, but not on the final amount of encapsulated active [137]. Of note, PEF has been shown to allow the encapsulation of water-soluble dyes [138] and large macromolecules (700 kDa FITC-dextrane [139]) in yeast.

For what attains to the application of negative pressure (vacuum) to yeast encapsulation, this approach also aims to increase the permeability of cell walls (e.g., via vesiculation); there appears to be only one report on the matter, where Young et al. employed two moderately hydrophobic compounds (fisetin, curcumin) for a combined encapsulation into either intact cells (more efficient encapsulation) or YCWs [140].

**Presence of water.** A sufficient level of yeast hydration appears to be critical, both for encapsulation and release. While evidence for the former is mostly anecdotical (e.g., encapsulation of limonene in a powder process does not work unless some water is present [94]); examples of the latter are more common: Dardelle et al. demonstrated that a minimum of 20% hydration is necessary for limonene release [32], while Dimopoulous et al. highlighted the need for water activity (a_w_) > 0.7 to obtain release [137], which tallies with the insensitivity of the loaded yeast cells to the % of humidity [27]. There is no clear consensus in relation to which element is more in need of hydration, but sparse evidence seems to suggest that both wall and membrane require it. For example, spray-dried, glucan-extracted *S. cerevisiae* vortexed into a hexane solution did not release a significant amount of hydrophobe (limonene, ethyl hexanoate) unless water was added [26]; since glucan extraction is an industrial process leading to cells with a reduced or no cell wall, but typically with a still functional membrane, it seems likely that water is necessary not only for the obviously critical swelling of the wall, but also to hydrate the membrane. It is worth mentioning that membranes can also be transiently permeabilized during (re)hydration: a sub-lethal hyperosmotic treatment (glycerol solutions with osmotic pressures of 1.4 and 30 MPa) during rehydration allows higher EE% thanks to the inward water flow, and this even allows the (non-endocytic, just purely diffusive) encapsulation of FITC-labelled dextran with a molar mass of 20 kDa [141]. 

**Size and polarity of the compound to encapsulate.** Although it may not be completely intuitive, dimensions and polarity are intimately related to the concept of permeation through a barrier. Permeation is indeed defined as the product of the solubility (or simply the concentration) of a species in a given environment multiplied by its diffusion coefficient, which is obtained from the hydrodynamic dimensions of the molecule weighted by the viscosity of the environment (Stokes–Einstein equation).

**The ‘sieving-out’ interpretation and its problems.** A commonly encountered interpretation of the way hydrophobes get (or not) into yeast invokes the porosity of the barrier structures as a critical attribute; their (tight) porosity should explain why only low molecular weight molecules (typically less than 700 g/mol) [142] can freely permeate. We will here refer to this as the ‘sieving out’ interpretation; according to this, since the cell wall acts as a sieve with very small pores, the process would be dominated by the size ratio between compounds to be encapsulated and the pore. In this interpretation, neither the absolute size of the compound (hence its diffusion coefficient) nor its solubility in the cell wall would play a major role.

This interpretation, however, fails to account for several experimental findings. First, it has been shown that 15 nm quantum dots can permeate through YCWs [65]; this can be allowed by partial degradation of the walls in the preparative process, but nonetheless is an indication that the mesh size of the glucan network is much larger than the molecular dimensions. Second, this ‘sieving out’ hypothesis does not explain why, for example, 1-decanol (C_10_H_21_OH; linear with a polar end) has a 4–5 times higher encapsulation rate than limonene (C_10_H_16_; cyclic, with unsaturation) and about 15 times higher than decane (C_10_H_22_; linear and only aliphatic), which are all similar in size; nor does it rationalize why EE% falls abruptly with alkanes even slightly larger than decane (undecane, dodecane) [42]. It is indeed difficult to imagine a mesh size so precise as to distinguish compounds based on a size difference of one carbon atom. Last, it has been shown that the reduction of disulfide bonds between mannoproteins—although in principle affecting the mesh size of the external part of the cell wall—produced no change in encapsulation [100,102] and most critically in its molecular weight threshold (the largest size a molecule can have to permeate [104]). 

**Permeation as a solubility-controlled phenomenon.** In contrast to the ‘sieving-out’ hypothesis, there are several pieces of evidence that solubility—both in the wall and in the membrane—is the key parameter to look at. Pham et al. analyzed the EY% of three groups of amphiphilic molecules, carboxylic acids (C_3_–C_8_), ethyl esters (C_4_–C_12_), and lactones (C_6_–C_12_), finding a logP (octanol/water partition coefficient) of about 1 as the minimum required to have a measurable encapsulation [102]. This general behavior has been confirmed by Degrelle et al., who showed that EE% could even exceed 50% when logP was higher than 2 [32]. However, in the study of Pham et al., a logP dependency was verified only within each group of compounds, but not between different groups; indeed EY% was higher for polar compounds (carboxylic acids and, surprisingly, lactones) than for apolar ones (esters). This is in accordance with the previously mentioned observation that decanol encapsulates much better than decane [42], and also by the observation of our group that encapsulation (at several temperatures) was progressively slower and lower in amount passing from carvone (log P = 2.2) through linalool (log P = 3.6) to limonene (log P = 4.6) [31]. Therefore, it seems logical to conclude that one needs an all-in-all hydrophobic character (logP > 1), most likely to ensure solubility in and thus permeation through the cell membrane, but also a distinct solubility in a water-based environment such as the cell wall.

It must also be noted that the above considerations focused predominantly on the enthalpic contributions to solubility (i.e., how hydrophobic a compound is). There are, however, also entropic contributions, which are mostly influenced by its molecular weight.

**Solubility is not only controlled by interactions (enthalpy).** There are also entropic contributions to the solubility of a compound in a given environment; these contributions can help to rationalize why molecules with comparable hydrophilicity (i.e., similar $\Delta H_{sol}$) but different size may permeate differently [104], without necessarily invoking a ‘sieving-out’ effect.

Using a rough approximation, we can assimilate the variation of entropy associated with the solubilization of a compound *x* in the cell wall ($\Delta S_{sol}$) to that seen in its dissolution in water. According to the Boltzmann equation, $\Delta S_{sol}=-k_{B}\left(n_{H_{2}O}\ln\phi_{H_{2}O}+n_{x}\ln\phi_{x}\right)$, where $\phi_{H_{2}O}$ and $\phi_{x}$ are the volume fractions of water and of the compound, and $n_{H_{2}O}$ and $n_{x}$ are their respective number of molecules. If the size of *x* is significantly larger than that of water, the volume units (=lattice sites) $N_{x}$ occupied by each of its molecules will be significantly larger than that occupied by a molecule of water $N_{H_{2}O}$ or any other low molecular weight compound, which by convention is typically equated to 1; correspondingly, $N_{x}$ becomes a measure of the size of *x*, for example, its degree of polymerization, or the number bases for a nucleic acid, etc. The last expression above can be reworked as $\Delta S_{sol}=-k_{B}n_{tot}\left(\frac{\phi_{H_{2}O}}{N_{H_{2}O}}\ln\phi_{H_{2}O}+\frac{\phi_{x}}{N_{x}}\ln\phi_{x}\right)\propto -(\phi_{H_{2}O}\ln\phi_{H_{2}O}+\frac{\phi_{x}}{N_{x}}\ln\phi_{x})$, where the second contribution is inversely proportional to the size of the molecule *x*, and therefore becomes negligible (e.g., for polymers). In short, the lack of permeation for large molecules can be naturally explained on thermodynamic grounds (vanishing entropic contribution, possibly low or no enthalpic gain), instead of invoking their ‘sieving out’. Clearly, the overall process will have both enthalpic and entropic contributions; for example, using an enthalpically more favorable environment (e.g., replacing water with DMSO in the cell wall) may allow permeation of compounds that under other conditions are (entropically) too large to permeate [104].

## 5. Mechanistic Considerations

**Figure 4:**
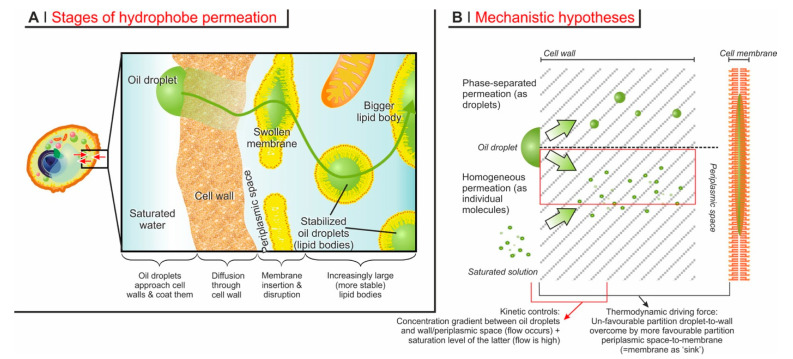
(**A**). The process of diffusional encapsulation of hydrophobes into an (intact) yeast cell can be broken down into adsorption on the wall, permeation through the cell wall and periplasmic space, penetration in the cell membrane, and finally the disruption of the latter (and of all other membrane-limited organelles) to form large lipid bodies. (**B**). Mechanistically, it could be hypothesized that the hydrophobes permeating through cell wall do so both from adsorbed droplets and from the (saturated) external solution, or that they pass the membrane as small hydrophobic aggregates or as individual molecules. Experimental evidence and simple considerations lead to the conclusion that the hydrophobes should permeate as individual molecules originating from oil droplets (highlighted in the red box). Furthermore, there are two main partition equilibria: from adsorbed droplets to the cell wall, and from the periplasmic space to a (swollen) membrane/lipid body; the stabilization offered by the cell surfactants to the latter is likely the main thermodynamic driving force of the whole process.

The process of encapsulation may be thought to articulate in four successive phases (Figure 4A).

**The approach to the cell wall (adsorption).** Most compounds of interest are scarcely soluble in water, therefore, in the encapsulation environment, they form oil droplets of variable size depending on the details of the process. It is reasonable to assume that the oil droplets adsorb on the surface of the cells before the hydrophobes migrate into the cell body; this phenomenon has been reported for the oil-degrading yeast *Yarrowia lipolytica* [143]. It has also often been observed in practice—but seldom reported in papers—that clusters of cells form during encapsulation (see e.g., Figure 5 in [92]), which is likely a consequence of ‘oily’ layers on their surfaces. An important observation is that most processes do not make use of surfactants, which means that the adsorption on a yeast surface would help to reduce the otherwise high surface energy of oil droplets; indeed it has been shown that the adsorption of hydrophobes on yeast surfaces is exothermic, reportedly with a maximum efficiency below 35 °C [144]. Even so, extensive description and sound experimental evidence of this phenomenon is still lacking.

From a physico-chemical point of view, adsorption on yeast surfaces has been predominantly studied in the context of water detoxification from heavy metal ions [145,146]) and also a bit less commonly from dyes [145,147] and pollutants [148]; for an exhaustive list of adsorbates, the reader is referred to the review of Aksu [149], and, in short, it is safe to say that this phenomenon is relatively well known for water-soluble species. A thermodynamic description of the adsorption of hydrophobic species is, in contrast, exceedingly complex: there are plenty of kinetic (non-equilibrium) issues in the fluidodynamics of the system and the mass transfer of the droplets that hinder both the creation of a meaningful model and its experimental validation. 

**Permeation through the cell wall.** This stage is often—and probably rightly—considered to be the rate-determining step of encapsulation [136]. Since no transport proteins have been detected in cell walls [150], this stage is typically considered to be based on passive diffusion through water-swollen pores. 

In principle, three mechanisms may be operational: diffusion through cell wall pores of individual molecules originating from adsorbed droplets, or migrating from a saturated solution, or migration of conspicuous, phase-separated amounts (e.g., oil droplets) of hydrophobes (Figure 4B). The latter mechanism, however, can be easily discounted, based on the following considerations.

- In the previous section, we discussed how the permeation through the complex barrier comprising both wall and membrane should be interpreted as predominantly determined by their solubility in both environments. This would not apply if the hydrophobes permeate as oil droplets, since effectively, their molecules would not be solubilized;
- In a previous study [31], we demonstrated that when a low molecular weight compound (limonene) was mixed in variable amounts with a short polymer (a polysulfide), the encapsulation of the two in mixture was similar to when they were employed individually (i.e., high for limonene and low for the polysulfide); furthermore, this was irrespective of their ratio, and therefore also independent of the viscosity of the putative oil droplets; and
- The molecular weight threshold discussed in the last section can only be thought of in the context of the permeation of individual molecules.

As discussed in Ciamponi et al. [31], it is also possible to discount the permeation originating from the hydrophobe’s external saturated solution. The driving force for diffusion through the cell wall is a gradient in concentration of the hydrophobe across it. This is the maximum when on one side there is an oil droplet (maximum achievable concentration) and on the other the periplasmic space, even when the latter becomes saturated. In contrast, there would be hardly any gradient between the external solution and periplasmic space. 

In short, all of the available evidence suggests that the diffusional process be based on the permeation of individual molecules from oil droplets through the cell wall.

**‘Permeation’ through the cell membrane.** Little is known regarding the conditions of the periplasmic space, but it is reasonable to assume that, bar some possible enzymatic degradation, molecules coming out the cell wall have their next barrier in the cell membrane. Since encapsulation is highest for compounds with logP 2–3, which therefore dissolve better in a lipidic environment, the cell membrane will necessarily act as a ‘sink’ for them (i.e., an environment that stabilizes them thermodynamically), and therefore drives the diffusion in a specific direction. At some point, however, the incorporation of increasing amounts of hydrophobes will necessarily disrupt the integrity of the membrane; indeed, no recognizable cell membrane exists in loaded YBMCs, and its components are likely to be found on the surface of the newly formed large lipid bodies. Thermodynamically, membrane loss of integrity changes very little: there are plenty of other membranous structures in the yeast cytoplasm that can replace the plasma membrane as ‘sinks’ for the exogenous hydrophobes. Kinetically, the disappearance of the closest membrane to the cell wall may decelerate encapsulation: the hydrophobes would need to cover a larger distance (the wider ‘periplasmic’ space seen in Figure 3) before finding a ‘sink’. Interestingly, in Ciamponi et al., we recorded ‘biphasic’ kinetics: a rapid (<10 min) phase was followed by a slower (up to hours to complete) one, and the majority of the hydrophobe appears to have been encapsulated in the latter; a possible—although maybe simplistic-interpretation—would be that the rapid phase may correspond to the swelling of the cell membrane until its disruption, and the slower phase to the distribution of the hydrophobes in the variety of lipid bodies generated by the cytoplasm reorganization following the membrane collapse.

In summary, (A) the experimental evidence is far from sufficient to confirm any mechanistic hypothesis; (B) yet, it seems reasonable to think that, rather than simply permeating through the cell membrane, hydrophobes would first accumulate in it, then would disrupt it, and finally distribute in all other lipidic environments within the cell.

## 6. Conclusions

Yeast cells can be successfully turned into microcapsules. In the first part of this review, we discussed the structurally different types of yeast-based microcontainers present in the recent literature. Although with significant differences among them, passive diffusion is the standard encapsulation process, which has also been used for commercial purposes; the physico-chemical factors controlling it, however, have been comparatively less investigated. Here, we specifically focused on the passive permeation in intact (or at most permeabilized) cells, discussing its different phases (adsorption, permeation through cell wall, interactions/disruption of cell membrane) in qualitative terms. Unfortunately, a more detailed, quantitative analysis and the development of predictive models are still hampered by a significant lack of standardized procedures, with variables including the yeast type, the details of its culture, the fluidodynamics of the microencapsulation process, and the conditions of any post-encapsulation treatment and storage.

## Data Availability

Not applicable.
